# The contribution of health systems research to HSR: time to know what we are talking about, and why it is important for evidence-based policy-making

**DOI:** 10.1186/1472-6963-14-S2-O25

**Published:** 2014-07-07

**Authors:** Reinhard Busse

**Affiliations:** 1 Department of Health Care Management, Technische Universität Berlin & European Observatory on Health Systems and Policies, Germany

## 

Within health services research (HSR), health systems research addresses the macro level of health care, i.e. the level of nations or regions, and the issues related to the organization and governance of the system, its model of financing, the ways to create physical and human resources, the provision of services, as well as its changes over time.

According to the WHO Tallinn Charter a health system is “... the ensemble of all public and private organizations, institutions and resources mandated to improve, maintain or restore health. Health systems encompass both personal and population services, as well as activities to influence the policies and actions of other sectors to address the social, environmental and economic determinants of health”. This is only one of many definitions used around the world. Although there is strong global consensus on the need to strengthen health systems, there is no consensus on an established framework or a common definition that could help students, researchers, policy-and decision-makers in studying, comparing, reforming and analyzing health systems. This leads to misunderstandings and different interpretations about scope, transferability of experiences, functions and goals; failed interventions with undesired side effects; and flawed assessments and feedback.

This is problematic particularly also because health systems have become increasingly complex and are rapidly changing. Moreover, health systems have to address important macro level challenges, which may vary greatly around the globe. Whereas a low-income country may be faced with low health spending, limited capacity to raise revenue, a rapidly growing population and lack of access to care of good quality, a typical high-income country could be struggling with unsustainable growth in health spending, a rapidly ageing population and increasing OOP. However, both have a similar challenge: how to best utilize scarce resources. It is therefore of great value to recognise and adopt organizational and technical innovations that can help make health care more effective and efficient. This calls for more understanding and more empirical evidence on both intended and unintended consequences of different types of action on health systems.

Therefore, it is time for a common concept of the many elements of health systems, the dynamics between these elements, the different approaches chosen around the world - and last but not least, how to research and evaluate these in order to provide an evidence base for better policy-making. The backbone of the presentation will be a simplified triangular model of a health system, outlining the main players and their interactions, which is particularly suitable to systematically describe, compare and analyze the arrangements used in health systems.

